# Systematic Review of Surgical Approaches for Adrenal Tumors: Lateral Transperitoneal versus Posterior Retroperitoneal and Laparoscopic versus Robotic Adrenalectomy

**DOI:** 10.1155/2014/918346

**Published:** 2014-12-17

**Authors:** Young Jun Chai, Hyungju Kwon, Hyeong Won Yu, Su-jin Kim, June Young Choi, Kyu Eun Lee, Yeo-Kyu Youn

**Affiliations:** ^1^Department of Surgery, Seoul National University Boramae Medical Center, 20 Boramae-ro 5-gil, Dongjak-gu, Seoul 156-70, Republic of Korea; ^2^Cancer Research Institute, Seoul National University College of Medicine, 101 Daehak-ro, Jongno-gu, Seoul 110-744, Republic of Korea; ^3^Department of Surgery, Seoul National University Hospital and College of Medicine, 101 Daehak-ro, Jongno-gu, Seoul 110-744, Republic of Korea; ^4^Department of Surgery, Seoul National University Bundang Hospital and College of Medicine, 300 Gumi-dong, Bundang-gu, Seongnam 463-707, Republic of Korea

## Abstract

*Background*. Laparoscopic lateral transperitoneal adrenalectomy (LTA) has been the standard method for resecting benign adrenal gland tumors. Recently, however, laparoscopic posterior retroperitoneal adrenalectomy (PRA) has been more popular as an alternative method. This systematic review evaluates current evidence on adrenalectomy techniques, comparing laparoscopic LTA with PRA and laparoscopic adrenalectomy with robotic adrenalectomy. *Methods*. PubMed, Embase, and ISI Web of Knowledge databases were searched systematically for studies comparing surgical outcomes of laparoscopic LTA versus PRA and laparoscopic versus robotic adrenalectomy. The studies were evaluated according to the PRISMA statement. *Results*. Eight studies comparing laparoscopic PRA and LTA showed that laparoscopic PRA was superior or at least comparable to laparoscopic LTA in operation time, blood loss, pain score, hospital stay, and return to normal activity. Conversion rates and complication rates were similar. Six studies comparing robotic and laparoscopic adrenalectomy found that outcomes and complications were similar. *Conclusion*. Laparoscopic PRA was more effective than LTA, especially in reducing operation time and hospital stay, but there was no evidence showing that robotic adrenalectomy was superior to laparoscopic adrenalectomy. Cost reductions and further technical advances are needed for wider application of robotic adrenalectomy.

## 1. Introduction

Open transperitoneal adrenalectomy has been the gold standard of treatment for adrenal disease. According to the Nationwide Inpatient Sample [1], 83% of adrenalectomies from 1998 through 2006 were performed using the open method, despite laparoscopic adrenalectomy shown to be successful in 1992 [2]. As conventional open adrenalectomy offers a wide surgical view and operative field, it is still preferred to laparoscopic adrenalectomy for large tumors and malignancies [3]. Laparoscopic adrenalectomy is not indicated for malignancy because it is associated with higher recurrence rates [4], although it could be performed safely in selected patients with isolated metastatic adrenal tumors [5].

More recently, however, laparoscopic procedures have been shown to be advantageous, with minimally invasive adrenalectomy replacing open adrenalectomy. Minimally invasive adrenalectomy results in less blood loss, earlier ambulation, shorter hospital stay, and faster return to normal activity [6]. These advantages were made possible by accumulated experience, advanced laparoscopic techniques, and better understanding of adrenal gland anatomy. At present, lateral transperitoneal adrenalectomy (LTA) has become the most widely utilized procedure for patients with benign adrenal disease. Other minimally invasive techniques include the anterior transperitoneal approach and the retroperitoneal (lateral or posterior) approach [7].

Since its introduction in 1995, posterior retroperitoneal adrenalectomy (PRA) has been utilized more frequently [8]. This technique consists of approaching the adrenal gland directly through the retroperitoneal space, while not breaching the peritoneum, resulting in a shorter operative time, less blood loss, less postoperative pain, and shorter hospital stay [9].

Although several studies have compared the outcomes of LTA and PRA, their overall results are inconclusive. Each study was retrospective in design and was conducted in a single center with different inclusion criteria. In addition, several meta-analyses have compared transperitoneal with retroperitoneal adrenalectomy [9–11]. These analyses, however, were not fully reliable, because the numbers of included studies and the study population were insufficient for meta-analyses. Furthermore, these analyses included older studies, which utilized different operation methods than those used currently or did not distinguish true PRA from lateral retroperitoneal adrenalectomy. This systematic review therefore compared surgical outcomes in patients undergoing LTA or PRA, as well as comparing outcomes in patients undergoing robotic or laparoscopic LTA or PRA.

## 2. Methods

### 2.1. Literature Search

The PubMed, Embase, and ISI Web of Knowledge databases were searched for studies, published in English after 2000, comparing laparoscopic LTA with PRA and robotic adrenalectomy with laparoscopic adrenalectomy. Using the criteria of the PRISMA statement, two authors (Young Jun Chai and Hyungju Kwon) independently searched the literature on 25 May 2014 for relevant studies [12]. Keywords for comparisons of laparoscopic LTA with included “lateral transperitoneal adrenalectomy,” “laparoscopic transabdominal adrenalectomy,” “laparoscopic transperitoneal adrenalectomy,” “retroperitoneoscopic adrenalectomy,” and “retroperitoneal adrenalectomy.” Keywords for comparisons between robotic and laparoscopic LTA or PRA included “robotic adrenalectomy” and “robot-assisted adrenalectomy.” References cited in relevant papers were also searched manually.

### 2.2. Inclusion and Exclusion Criteria

Clinical studies comparing LTA with PRA or laparoscopic with robotic adrenalectomy were reviewed by the two independent authors. Only single center studies that enrolled more than 20 patients were included. After reviewing operative methodology, studies that used the lateral retroperitoneal approach were excluded, allowing comparisons of LTA with true PRA. Studies that did not describe surgical procedures in detail, enabling categorization as LTA or PRA, were also excluded. In comparisons of laparoscopic and robotic adrenalectomy, studies that did not report the outcomes of LTA and PRA separately were excluded. If there were multiple reports from the same study patients, only the most recent or the largest study was included.

### 2.3. Data Extraction

Data collected by the two independent authors included the last name of the first author, study design, year of publication, patient characteristics, indications for LTA or PRA, operation time, blood loss, pain score, hospital stay, conversion rate, and characteristics of complications. Conversion to open surgery and conversion from robotic to laparoscopic surgery were counted together.

### 2.4. Surgical Procedures of Laparoscopic LTA

For LTA, the patient is placed in the lateral decubitus position with the affected side facing upward and the operative bed flexed just above the level of the iliac crest [13]. In general, three ports are made for left adrenalectomy, with one additional port required for right adrenalectomy to mobilize and lift the liver. Although the ports were commonly made at the umbilicus and the subcostal area in the anterior axillary and midclavicular lines, port sites could be modified at the discretion of the surgeon. All ports were inserted through the peritoneum, with the adrenal gland exposed by dissecting adjacent organs. The spleen, distal pancreas, and splenic flexure of the colon were detached from the retroperitoneum for left adrenalectomy, whereas the triangular ligament of the liver was dissected and rotated medially using a fan retractor for right adrenalectomy. After dividing the surrounding blood vessels, including the adrenal vein, the adrenal gland was placed in a plastic bag and retracted through a port site.

### 2.5. Surgical Procedures of Laparoscopic PRA

This procedure was performed as described [14]. Briefly, anesthesia was induced in a patient bed, and the patient was intubated and turned from the bed onto the operating table. The patient was placed in a prone position, lying on the abdomen with bent hip and knee joints. In general, three ports were required for this procedure. The first incision, about 1.5 cm in size, was made just below the tip of the twelfth rib, and the retroperitoneal space was bluntly dissected with a finger. Through this space, the second and third ports were made under direct vision 4-5 cm medially and laterally. After CO_2_ insufflation, perinephric fatty tissues were dissected from the posterior aspect of kidney, and the superior pole of the kidney was exposed. The inferior aspect of the adrenal gland was mobilized from the superior pole of the kidney. Adrenalectomy was completed by detaching the adrenal gland from adjacent structures and ligation of the adrenal vein. The resected adrenal gland is placed in a plastic bag and pulled out through the first incision site.

### 2.6. Procedures of Robotic LTA and Robotic PRA

Robotic adrenalectomy was firstly performed in 2000 [15]. The surgical procedures of robotic LTA and PRA are basically similar to those of laparoscopic LTA and PRA, such as patient position, port sites, use of CO_2_ gas, and specimen removal [16, 17]. The patient is placed in a lateral decubitus position with lesion side upward (LTA) or in the prone jackknife position with bent hip and knee joints (PRA). After creation of pneumoperitoneum, to be used as a 12 mm camera port, the other two or three 5 mm robotic ports are made. The robot is docked, with subsequent procedures basically identical to those of laparoscopic LTA and PRA.

### 2.7. Statistical Analysis

Results were analyzed with SPSS version 19 (SPSS, Inc., Chicago, IL, USA). Mean and median values with standard deviation were used for numeric data. Univariate analysis was performed using the Mann-Whitney *U* test, chi-Square test, or Fisher's exact test, as warranted. *P* < 0.05 was considered statistically significant.

## 3. Results

**Figure 1:**
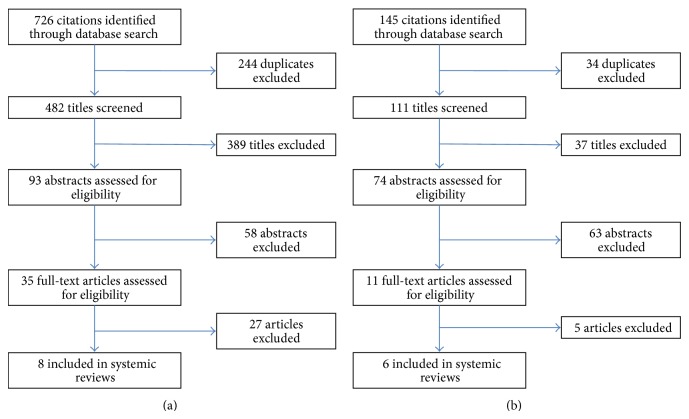
Flow charts showing selection of articles for systematic review. (a) Laparoscopic lateral transperitoneal adrenalectomy versus posterior retroperitoneoscopic adrenalectomy. (b) Laparoscopic adrenalectomy versus robotic adrenalectomy.

In comparing LTA and PRA, the literature search yielded 482 titles, after excluding duplicates (Figure 1). After screening for relevance, 93 abstracts were deemed eligible for review; of these, 58 were excluded as they were not original papers or did not meet inclusion criteria. Of the 35 full-text articles searched, eight were included in the analysis. In comparing laparoscopic adrenalectomy with robotic adrenalectomy, 111 titles were selected, after excluding duplicates. After screening the abstracts and full-texts, a total of six articles were included.

### 3.1. Indications and Contraindications for Laparoscopic LTA and PRA

Table 1 shows the size criteria and patient characteristics of the eight studies comparing laparoscopic LTA with true PRA [18–25]. In most of the studies, malignant tumors were considered a contraindication for laparoscopic surgery; however, LTA or PRA was successfully performed in patients with no evidence of invasion on preoperative imaging or when the lesion was an isolated metastasis [20, 21]. Patients were deemed eligible for PRA if their tumors were less than 8 cm in diameter, although size criteria were more liberal for LTA. There were no significant age differences in selecting LTA or PRA, but some studies reserved PRA for patients with lower body mass index (BMI) and smaller tumor size [20, 21, 24].

### 3.2. Comparative Surgical Outcomes: Laparoscopic LTA versus Laparoscopic PRA

The surgical outcomes of patients undergoing LTA and PRA are shown in Table 2. PRA and LTA showed similar operation times in early studies [18–21], although more recent studies reported that operation time was shorter for PRA than for LTA [22–24]. Four studies described intraoperative blood loss [18, 20, 22, 23], with one finding that blood loss was lower with PRA [22], and the other three reported no significant difference. Postoperative pain score was compared in one study [21], which found that pain scores on postoperative days one and three were significantly lower in the PRA than in the LTA group. Seven studies compared hospital stay after operation with the five most recent studies finding that mean hospital stay was shorter in patients who underwent PRA than LTA [21–25]. Time to return to work or days of convalescence were evaluated in two studies [18, 19], with one showing that PRA was associated with earlier return to normal activity [19], and the other reporting similar results in patients undergoing PRA and LTA [18].

### 3.3. Complications: Laparoscopic LTA versus Laparoscopic PRA

Complications of laparoscopic LTA and PRA are summarized in Table 3. Two studies reported conversion to open surgery or conversion from PRA to LTA [18, 20]. In one, four patients were converted to open surgery from LTA and three from PRA. Of them, excessive operation time was the cause in 3 LTA and in 2 PRA cases [18]. One patient who underwent LTA required conversion due to a diaphragm injury and one who underwent PRA required conversion due to intercostal artery bleeding caused by trocar insertion. In the other study, two patients were converted to open surgery from LTA due to bleeding from the adrenal vein, and two were converted from PRA to LTA due to inadequate establishment of retroperitoneal space during the initial learning curve for PRA [20]. Total conversion rates were 2.3% (6/263) for LTA and 1.9% (5/265) for PRA.

Two patients who underwent LTA died from cardiac and pulmonary complication [20], making the mortality rates 0.7% and 0% in patients undergoing LTA and PRA, respectively. Postoperative bleeding (1.1%; 3/263), diaphragm injury (0.4%; 1/263), pulmonary embolism (0.8%; 2/263), port site incisional hernia (0.4%; 1/263), postoperative ileus (0.4%; 1/263), pneumonia (0.4%; 1/263), and major cardiac arrhythmia (0.4%; 1/263) occurred only in the LTA group. Complications in patients undergoing PRA included thrombophlebitis (0.4%; 1/265), temporary hypoesthesia (0.4%; 1/265), neuralgia (0.8%; 2/265), paresthesia (0.4%; 1/265), lateral abdominal swelling (1.5%; 4/265), pneumothorax (0.4%; 1/265), pleural effusion (0.4%; 1/265), and stroke (0.4%; 1/265). Wound infection occurred in one patient each undergoing LTA and PRA (0.4% each). Total complication rates were 5.3% (14/263) for LTA and 5.3% (14/265) for PRA.

### 3.4. Comparative Surgical Outcomes: Laparoscopic versus Robotic Adrenalectomy

Since the introduction of the da Vinci surgical robot system (Intuitive Surgical, Inc., Sunnyvale, CA, USA) for cardiac surgery in 1998, its use has expanded to many other types of surgery, including endocrine surgery. The robotic system was found to overcome some of the limitations of laparoscopic surgery, including its inflexibility, two-dimensional operative view, and fatigue, by providing a tremor-free endowrist, three-dimensional imaging, and excellent ergonomics [26, 27]. Despite its high cost, many surgeons have applied robotic surgery to LTA and PRA to maximize surgical efficiency.

Six studies met the inclusion criteria; their results were shown in Table 4 [28–33]. During the early period of robot use, as shown in the first two studies, operation times were significantly longer for robotic LTA than for laparoscopic LTA [28, 29]. More recently, however, as shown in the four later studies, there were no differences in operation time between laparoscopic and robotic LTA [30–33]. Two studies reported that blood loss was lower for robotic LTA [29, 33], and one study reported less pain on postoperative day one for robotic LTA [30]. Hospital stay was similar in both groups, although one study reported shorter hospital stay in the robotic group [31].

### 3.5. Complications: Laparoscopic versus Robotic Adrenalectomy

Table 5 shows complications of laparoscopic and robotic adrenalectomy. The most common cause of conversion from robotic to laparoscopic adrenalectomy or open laparotomy was bleeding (2.2%; 4/186). Other causes included inadequate visualization (1.6%; 3/186), prolonged operation time (0.5%; 1/186), and tumor adhesion (0.5%; 1/186). Patients with complications, such as bleeding or tumor invasion, were converted to open laparotomy, whereas those with minor problems, such as inadequate visualization, were converted to laparoscopic procedures. Overall conversion rates were similar for robotic and laparoscopic adrenalectomy.

There were no unique complications related to robotic adrenalectomy. Types of complications in patients undergoing robotic procedures included pneumonia (1.6%; 3/186), wound problems (1.6%; 3/186), urinary tract infection (0.5%; 1/186), postoperative ileus (0.5%; 1/186), chylous ascites (0.5%; 1/186), hyponatremia (0.5%; 1/186), vomiting (0.5%; 1/186), atrial fibrillation (0.5%; 1/186), and postoperative bleeding requiring blood transfusion (0.5%; 1/186).

## 4. Discussion

The present systematic review suggests that PRA may be superior to LTA in terms of shorter operation time and hospital stay, as well as reduced blood loss and pain score. The superiority of PRA may be due to the location of the adrenal glands in the posterior aspect of the retroperitoneum. PRA directly approaches this space, enabling adrenalectomy without collateral damage to adjacent intra-abdominal organs, which must be dissected and mobilized during LTA or open adrenalectomy [34]. In consequence, recovery of bowel movement was faster and postoperative ileus rarely observed in the patients who underwent PRA. Furthermore, PRA is feasible in patients with a previous history of abdominal surgery, as well as being suitable for bilateral adrenalectomy. Reports of good intra- and postoperative outcomes of PRA have made this method more popular. However, PRA is not easy for beginners to learn because surgeons are not familiar with this anatomic view of the retroperitoneal space. Thus training in the use of PRA requires a substantial amount of time. PRA is also difficult to perform in patients with tumors larger than 7-8 cm and in patients with a high BMI [14].

In three of the eight studies enrolled in this review, operation time was significantly shorter for laparoscopic PRA than LTA; the other five studies showed no between group differences in operation times. The shorter operation time in PRA was most likely due to the smaller extent of dissection required. Moreover, PRA was performed by surgeons skilled in the laparoscopic technique for LTA, allowing them to overcome the learning curve for PRA more easily. A comparison of the first 50 operations performed by the developers of PRA and 50 operations performed by a surgical team that learned PRA from the developers found that the learning curve was shorter in the latter group, suggesting that comprehensive training resulted in a shorter operation time and a lower conversion rate [35]. Hence, PRA is safe when performed by surgeons with LTA experience or those who undergo proper PRA training.

In all five studies reported since 2011, hospital stay was shorter in the PRA than in the LTA group [21–25], in agreement with the results of a meta-analysis comparing LTA and PRA [9]. Shorter hospital stay may be associated with reduced pain, as the median visual analog pain scores in patients undergoing PRA and LTA were reported as 0 and 3, respectively [21]. Another parameter indicating the amount of postoperative pain is analgesic use during hospitalization, which was evaluated in three studies [19, 23, 25]. Analgesic use was lower in the PRA group in two of these studies [23, 25] and equal in the third [19].

Conversion rates of LTA and PRA to open laparotomy were 2.3% (6/263) and 1.9% (5/265), respectively, but the reasons for conversion differed. Conversion in LTA was associated with diaphragm injury or vascular injury, whereas conversion in PRA was associated with inadequate preparation of the retroperitoneal working space due to surgeon inexperience. A unique complication associated only with PRA was neuromuscular pain related to subcostal nerve injury [36]. The subcostal nerve passes below the twelfth rib and the injury to this nerve commonly occurs during open posterior adrenalectomy, leading to chronic incision-related back pain [37]. Likewise, trocar insertion in this area during PRA can cause nerve damage. The incidence of nerve damage in the largest PRA series was reported to be 9%, but was temporary in most patients [14]. Although concerns have been raised regarding the high CO_2_ pressure required for PRA [38], there were no complications associated with high pressure, except for air embolism in one patient causing a stroke [24]. In particular, even in the study comparing LTA with PRA in patients with pheochromocytoma, there were no significant hemodynamic differences, such as intraoperative hypertension, hypotension, and vasoactive medication use [22].

Early comparisons of robotic and laparoscopic LTA found that operation time was longer for the former [28, 29]. However, operation time is largely dependent on the surgeon's experience. A detailed analysis of operation time in 50 patients undergoing robotic and 59 undergoing laparoscopic LTA found that the learning curve for robotic LTA was 20 cases, with no difference after the learning curve between operation times for robotic and laparoscopic LTA [29]. Moreover, previous experience with laparoscopic LTA or assistance from a staff surgeon contributed to reductions in operation time. Another report evaluating 100 robotic adrenalectomies found that surgeon's experience and first assistant level were independent predictors of longer operation time [16]. Notably, operation time of robotic LTA was not associated with high BMI (>30 kg/m^2^) or large tumor size (>55 mm), whereas operation times of laparoscopic LTA were increased under those conditions. In comparative studies published since 2012, there were no significant differences in operation times between robotic and laparoscopic adrenalectomy [30–33]. A comparison of robotic and laparoscopic PRA showed that pain score on postoperative day 1 was significantly lower in the robotic group (2.5 versus 4.2, *P* = 0.008) [30]. The lower pain score in the robotic PRA group was attributed to reduced manipulation of the incision, with fewer instrument changes and less pressure exerted by the surgical team on the patient's back. However, the mean pain scores became similar on postoperative day 14.

Robotic surgery with a similar operation time and ergonomic improvements relative to laparoscopic surgery is attractive for surgeons, since robotic procedures offer more delicate dissection using endowrist and a magnified view. Robotic adrenalectomy may have advantages in maintaining a vascularized remnant during cortical sparing adrenalectomy [39]. However, it is unclear whether the substantial additional cost of the robotic procedure ($3,466 versus $2,737) is justified, since outcomes are equivalent [28]. No randomized controlled trial has compared robotic adrenalectomy with laparoscopic adrenalectomy, and no study to date has shown that robotic adrenalectomy is superior to laparoscopic adrenalectomy in postoperative outcomes. Thus, the costs of the initial purchase of the robot and instruments, as well as its maintenance, are obstacles to the expansion of robotic adrenalectomy.

Several promising technical applications can be easily adapted for robotic adrenalectomy. For example, after indocyanine green dye injection, robotic partial adrenalectomy can be performed safely using the da Vinci SI system with FireFly scopes, a light source, and a software upgrade [40]. Indocyanine green dye can easily delineate the tumor margins, allowing uninvolved adrenal tissue to be saved. Other promising applications include telemonitoring and telerobotic surgery. Telemonitoring may be effective method in performing a new surgical technique, allowing remote monitoring by a trained surgeon [41]. Robotic surgery is more suitable for telemonitoring because robotic systems offer three-dimensional images, and the monitoring surgeons can better view patient anatomy. Robotic adrenalectomy can also be better optimized for new technical advances such as telesurgery or image-guided surgery and promising surgical modalities.

## 5. Conclusions

PRA may be superior to LTA, as shown by shorter operation times and hospital stay, and can be performed safely after surgeons overcome the learning curve. Randomized controlled trials comparing these two techniques are necessary to objectively evaluate them, excluding selection bias and bias related to differences in surgeons' experiences with these techniques. Current data suggest that robotic adrenalectomy is safe and feasible but have not shown definite advantages over laparoscopic adrenalectomy to date. Cost reductions or further improvements in surgical outcomes are necessary to expand the use of robotic adrenalectomy.

## Figures and Tables

**Table 1:** Characteristics and clinical information of the included studies.

Author	Year	Size criteria, cm		Number of patients		Mean age, years (±SD)			Gender ratio (male : female)			BMI, kg/m^2^(±SD)			Mean tumor size, cm (±SD)		
		LTA	PRA	LTA	PRA	LTA	PRA	*P* value	LTA	PRA	*P* value	LTA	PRA	*P* value	LTA	PRA	*P* value
Naya et al. [18]	2002^a^	6	6	28	22	48.4 ± 12.5	53.3 ± 7.0	N.S	8 : 20	10 : 12	N.S	22.2 ± 3.2	22.5 ± 3.3	N.S	N.A	N.A	N.A
Lombardi et al. [19]	2008^b^	6	6	38	38	45.2 ± 13.0	48.9 ± 11.9	N.S	8 : 30	8 : 30	N.S	N.A	N.A	N.A	3.3 ± 1.2	3.1 ± 0.9	N.S
Berber et al. [20]	2009^a^	14	7	69	90	52 ± 14	51 ± 14	N.S	25 : 44	43 : 47	N.S	32 ± 9	28 ± 6	0.005	4.4 ± 0.3	2.8 ± 0.1	<0.001
Kiriakopoulos et al. [21]	2011^b^	8	8	30	30	49.5^*^	53.0^*^	N.S	11 : 19	9 : 21	N.S	N.A	N.A	N.A	4.9^*^	3.8^*^	0.035
Dickson et al. [22]	2011^a^	N.A	N.A	23	23	42.0 ± 18.1	47.3 ± 16.1	N.S	7 : 16	11 : 12	N.S	26.1 ± 5.4	26.2 ± 6.6	N.S	4.0 ± 2.2	3.3 ± 1.8	N.S
Lee et al. [23]	2012^a^	8.5	7	26	17	53.4 ± 10.0	57.4 ± 13.9	N.S	7 : 19	6 : 11	N.S	24.5 ± 2.6	25.0 ± 3.2	N.S	3.9 ± 3.8	2.6 ± 1.6	N.S
Constantinides et al. [24]	2013^b^	N.A	N.A	36	35	49.5 ± 13.6	49.1 ± 13.6	N.S	13 : 22	15 : 21	N.S	26.8 ± 4.6	29.8 ± 5.5	0.033	4.1 ± 2.3	2.8 ± 1.3	0.033
Cabalag et al. [25]	2014	8	8	13	10	47^*^	61^*^	N.S	5 : 8	5 : 5	N.S	28^*^	28.9^*^	N.S	3.2^*^	3.5^*^	N.S

Values are expressed as mean with standard deviation, ^*^except median.

LTA: lateral transperitoneal adrenalectomy; PRA: posterior retroperitoneoscopic adrenalectomy.

^a^retrospective study.

^b^case-control study.

N.S: not significant; N.A: not available.

**Table 2:** Comparative outcomes of laparoscopic LTA versus laparoscopic PRA.

Author	Year	Mean operation time, min			Mean blood loss, mL			Pain score			Mean hospital stay, days			Return to normal activity		
		LTA	PRA	*P* value	LTA	PRA	*P* value	LTA	PRA	*P* value	LTA	PRA	*P* value	LTA	PRA	*P* value
Naya et al. [18]	2002	202 ± 74	221 ± 87	N.S	113 ± 204	192 ± 365	N.S	N.A	N.A	N.A	9.0 ± 3.3	9.5 ± 3.5	N.S	20.8 ± 5.9	21.9 ± 5.3	N.S
Lombardi et al. [19]	2008	135 ± 47	114 ± 47	N.S	N.A	N.A	N.A	N.A	N.A	N.A	6.2 ± 2.4	5.6 ± 2.1	N.S	59 ± 21	26 ± 17	<0.001
Berber et al. [20]	2009	157 ± 7	138 ± 6	N.S	35 ± 7	25 ± 6	0.05	N.A	N.A	N.A	N.A	N.A	N.A	N.A	N.A	N.A
Kiriakopoulos et al. [21]	2011	77.5^a^	90.0^a^	N.S	N.A	N.A	N.A	4^a,b^ 3^a,c^	1^a,b^ 0^a,c^	<0.001 <0.001	4^a^	2^a^	<0.001	N.A	N.A	N.A
Dickson et al. [22]	2011	144.8 ± 42.4	99.9 ± 23.3	0.0001	123.8 ± 204.3	8.4 ± 19.1	0.020	N.A	N.A	N.A	3.1 ± 1.4	1.9 ± 0.9	0.003	N.A	N.A	N.A
Lee et al. [23]	2012	108.3 ± 34.5	87.2 ± 27.6	0.042	74.8 ± 145.2	20.0 ± 41.7	N.S	N.A	N.A	N.A	5.9 ± 3.6	3.0 ± 1.4	0.003	N.A	N.A	N.A
Constantinides et al. [24]	2013	131.7 ± 46.6	86.3 ± 49.5	0.0002	N.A	N.A	N.A	N.A	N.A	N.A	3.5 ± 3.0	1.6 ± 0.9	<0.001	N.A	N.A	N.A
Cabalag et al. [25]	2014	105^a^	90^a^	N.S	N.A	N.A	N.A	N.A	N.A	N.A	2^a^	1^a^	<0.001	N.A	N.A	N.A

Values are expressed as mean with standard deviation, ^a^except median.

LTA: lateral transperitoneal adrenalectomy; PRA: posterior retroperitoneoscopic adrenalectomy.

^b^pain score on postoperative day 1.

^c^pain score on postoperative day 3.

N.S: not significant; N.A: not available.

**Table 3:** Complications of laparoscopic LTA versus PRA.

Author	Conversion, *n* (%)		Postoperative bleeding requiring surgery, *n* (%)		Mortality, *n* (%)		Others, *n* (%)	
	LTA	PRA	LTA	LTA	LTA	PRA	LTA	PRA
Naya et al. [18]	4^a^	3^b^	0	0	0	0	2	2
Lombardi et al. [19]	0	0	1	0	0	0	0	2
Berber et al. [20]	2^c^	2^d^	0	0	2^e^	0	0	2
Kiriakopoulos et al. [21]	0	0	0	0	0	0	0	4
Dickson et al. [22]	0	0	2	0	0	0	0	2
Lee et al. [23]	0	0	0	0	0	0	0	0
Constantinides et al. [24]	0	0	0	0	0	0	3	1
Cabalag et al. [25]	0	0	0	0	0	0	4	1

Total	6/263 (2.3)	5/265 (1.9)	3/263 (1.1)	0/265 (0)	2/263 (0.7)	0/265 (0)	9/263 (3.4)	14/265 (5.3)

LTA: lateral transperitoneal adrenalectomy; PRA: posterior retroperitoneoscopic adrenalectomy.

^a^open conversion due to diaphragm injury or excessive operation time, ^b^open conversion due to intercostal artery bleeding or excessive operation time, ^c^open conversion due to bleeding, ^d^conversion from PRA to LTA due to inadequate establishment of retroperitoneal space, and ^e^cardiac and pulmonary complication.

**Table 4:** Comparative outcomes of laparoscopic versus robotic adrenalectomy.

Author	Year	Approach	Number of patients		Mean tumor size, cm (range)			Mean operation time, min (range)			Mean blood loss, mL			Mean hospital stay, days (range)		
			LA	RA	LA	RA	*P* value	LA	RA	*P* value	LA	RA	*P* value	LA	RA	*P* value
Morino et al. [28]	2004^a^	LTA	10	10	3.1 (1.5, 6.0)	3.3 (1.4, 6.5)	N.S	115.3 (95, 155)	169 (136, 215)	<0.01	N.A	N.A	N.A	5.4	5.7	N.S
Brunaud et al. [29]	2008^b^	LTA	59	50	3.4 (1.0, 8.0)	2.8 (0.9, 7.5)	N.S	87 (50, 160)	104 (60, 180)	0.003	71	49	<0.001	6.3	6.9	N.S
Agcaoglu et al. [30]	2012^b^	PRA	31	31	3.0 ± 0.2	3.1 ± 0.2	N.S	165.7 ± 9.5	163.2 ± 10.1	N.S	35.6 ± 9.9	25.3 ± 10.3	N.S	1^*^	1^*^	N.S
Karabulut et al. [31]	2012^b^	LTA	32	32	3.6 ± 0.3	4.8 ± 0.4	0.03	160 ± 9	165 ± 10	N.S	41 ± 20	41 ± 10	N.S	1.5 ± 0.9	1.1 ± 0.3	0.006
		PRA	18	18	2.7 ± 0.3	2.3 ± 0.3	N.S	170 ± 15	166 ± 9	N.S						
You et al. [32]	2013^b^	LTA	8	15	2.8 (1.0, 4.5)	2.6 (1.0, 5.5)	N.S	183.1 (75, 270)	207.0 (120, 320)	N.S	N.A	N.A	N.A	6.7 (5, 9)	5.9 (4, 7)	N.S
Brandao et al. [33]	2014^b^	LTA	46	30	4.0^*^	3.0^*^	0.02	120^*^	120^*^	N.S	100^*^	50^*^	0.02	2.5^*^	2^*^	N.S

Values are expressed as mean with standard deviation, ^*^except median.

LA: laparoscopic adrenalectomy; RA: robotic adrenalectomy.

^a^prospective randomized controlled study.

^b^retrospective study.

N.S: not significant; N.A: not available.

**Table 5:** Complications of laparoscopic versus robotic adrenalectomy.

Author	Approach	Number of patients		Open or laparoscopic conversion, *n* (%)		Total complications, *n* (%)			Postoperative bleeding requiring surgery, *n* (%)			Mortality, *n* (%)		
		LA	RA	LA	RA	LA	RA	*P* value	LA	RA	*P* value	LA	RA	*P* value
Morino et al. [28]	LTA	10	10	0	4 (40.0)^a^	0 (0)	0 (0)	N.S	0 (0)	0 (0)	N.S	0 (0)	0 (0)	N.S
Brunaud et al. [29]	LTA	59	50	4 (6.8)^b^	4 (8.0)^c^	9 (15.3)	5 (10.0)	N.S	N.A	0 (0)	N.A	0 (0)	0 (0)	N.S
Agcaoglu et al. [30]	PRA	31	31	0 (0)	0 (0)	0 (0)	0 (0)	N.S	0 (0)	0 (0)	N.S	0 (0)	0 (0)	N.S
Karabulut et al. [31]	LTA	32	32	2 (6.2)^d^	1 (3.1)^e^	5 (10.0)	1 (2.0)	N.S	N.A	N.A	N.A	1 (2.0)	0 (0)	N.S
	PRA	18	18	0 (0)	0 (0)				N.A	N.A	N.A			
You et al. [32]	LTA	8	15	0 (0)	0 (0)	2 (25.0)	2 (13.3)	N.S	0 (0)	0 (0)	N.S	0 (0)	0 (0)	N.S
Brandao et al. [33]	LTA	46	30	1 (2.3)^f^	0 (0)	5 (10.9)	6 (20.0)	N.S	1 (2.1)	1 (3.3)	N.S	0 (0)	0 (0)	N.S

Total		204	186	7 (3.4)	9 (4.8)	21 (10.3)	14 (7.5)		2 (1.0)	2 (1.1)		1 (0.5)	0 (0)	

LA, laparoscopic adrenalectomy; RA, robotic adrenalectomy.

^a^conversion to open laparotomy due to malposition of robotic trocars (*n* = 2), bleeding (*n* = 1), and prolonged operation time (*n* = 1).

^b^causes of conversion to open laparotomy were not described.

^c^conversion to LA due to inadequate visualization (*n* = 1), and conversion to open laparotomy due to bleeding (*n* = 3).

^d^conversion to open laparotomy due to peri-adrenal invasion or inflammation.

^e^conversion to open laparotomy due to adhesion to renal hilum.

^f^cause was not described.

N.S: not significant; N.A: not available.
